# Linking layered corneal compactness to macroscopic biomechanics: *in vivo* assessment via integrated densitometry and dynamic deformation analysis

**DOI:** 10.3389/fbioe.2026.1745321

**Published:** 2026-05-07

**Authors:** Fuqi Deng, Huazheng Cao, Wenjing Gao, Caohui Xu, Li Li, Ping Lu, Yan Wang

**Affiliations:** 1 Clinical College of Ophthalmology, Tianjin Medical University, Tianjin, China; 2 Tianjin Key Lab of Ophthalmology and Visual Science, Tianjin Eye Institute, Tianjin Eye Hospital, Tianjin, China; 3 Nankai University Eye Institute, Nankai University Affiliated Eye Hospital, Nankai University, Tianjin, China; 4 School of Medicine, Nankai University, Tianjin, China; 5 Department of Ophthalmology, Beijing Friendship Hospital Affiliated to Capital Medical University, Beijing, China; 6 Shandong Lunan Eye Hospital, Linyi, Shandong Province, China

**Keywords:** biomechanics, cornea, corneal biomechanics, corneal densitometry, corneal structure

## Abstract

**Objective:**

To investigate *in vivo* correlations between corneal dynamic deformation properties and structural compactness via layered densitometry, clarifying the main contributions of the corneal anatomical layers to macroscopic biomechanics.

**Design:**

Cross-sectional study.

**Methods:**

In the study, a total of 221 eyes from 221 healthy participants were enrolled. Corneal deformation properties were measured by the air-puff Corvis ST. Scheimpflug images of the cornea were captured to examine 50-sublayer densitometry values using the caliper technique. According to the spatial patterns of corneal densitometry with depth, the whole cornea was then divided into multiple layers. Different layers were corresponding to different corneal structures (epithelium, Bowman membrane, anterior, middle, and posterior stroma, Descemet membrane, and, endothelium). In each layer, 19 predetermined data point (1, 6, 12 points on the central, paracentral, and peripheral zones, respectively) were taken out for analysis.

**Results:**

Layered densitometry analysis revealed distinct structural compactness patterns across seven corneal layers (epithelium to endothelium). Significant correlations emerged between densitometry values and dynamic biomechanical parameters, exhibiting strong layer-specific and regional dependencies. The anterior cornea (Bowman’s membrane and anterior stroma) showed the most robust associations: increased densitometry in these layers correlated positively with stiffness parameters (SPA1 [stiffness parameter in the first applanation], SPHC [stiffness parameter highest concavity]; central SPHC r = 0.3, *P* < 0.001) and negatively with deformation susceptibility metrics (DeflAmpMax [maximum deflection near highest concavity], IR [Integrated radius]; central IR r = −0.229, *P* < 0.001). These correlations weakened posteriorly and peripherally. Mid-stromal and posterior layers demonstrated a weak biomechanical relevance, while Descemet’s membrane and endothelium exhibited isolated regional associations.

**Conclusion:**

The study established the *in vivo* evidence linking layer-specific corneal compactness (densitometry) to macroscopic biomechanics, with anterior stromal compactness being the primary determinant of corneal stiffness parameter. Higher densitometry in anterior layers was associated with a stiffer corneal response, such as a higher SPA1 and a lower overall deformation amplitude. Descemet’s membrane’s densitometry-reflected structural state minimally modulates whole-corneal deformation. This densitometry-deformation framework may help elucidate microstructural alterations underlying corneal disease, surgery and biomechanical dysfunction, transcending conventional whole-cornea biomechanical assessments.

## Introduction

Recent studies have demonstrated associations between corneal biomechanics and several eye diseases, including keratoconus, glaucoma, and dry eye (Molero-Senosiain et al., 2021; Soltani et al., 2025; Kaya et al., 2022). These conditions pose significant risks to vision, ranging from progressive visual impairment and reduced quality of life to potential irreversible blindness if left unmanaged (Ma et al., 2018; Safa et al., 2022; Spierer et al., 2016). Furthermore, age-related alterations in ocular biomechanics and microstructure are critical considerations in surgical planning, such as intraocular lens fixation in older patients, highlighting the broader need to understand tissue structure-function relationships across the lifespan (Li et al., 2023; Reinehr et al., 2024; Wang et al., 2023). Corneal biomechanics is inherently complex, with distinct structural mechanics across different tissue layers. However, currently available clinical-level biomechanical assessments only measure the deformation of the cornea as a whole under applied forces, without truly characterizing its intrinsic mechanical properties (Wang et al., 2023). While tissue-level mechanical testing, such as tensile and inflation experiments, can be conducted *in vitro*, these approaches cannot replicate the *in vivo* conditions of the human eye. Consequently, they are unable to elucidate the relationship between corneal microstructure and biomechanics under natural physiological states.

Corneal densitometry was considered as an optical parameter (Shen et al., 2019). It derives from quantitative evaluation of corneal backscattered light through the analysis of image brightness represented in percentage of gray levels, usually based on the Scheimpflug technology (Takacs et al., 2011). In the traditional sense, corneal densitometry reflects the transparency of corneal tissue; it is used to quantify the extent of subepithelial corneal opacity and stromal scarring, as well as disease severity and healing status, including keratoconus and infectious keratitis (Takacs et al., 2011). However, fundamentally speaking, corneal densitometry reflects the tissue density and conveys information about its composition, correlated with collagen fiber alignment, hydration status, and intercellular spacing, thereby serving as a quantifiable indicator for evaluating microscopic material properties of the tissue (Yang et al., 2025; He et al., 2023). Previous studies have commonly assessed densitometry by dividing the cornea into anterior, central, and posterior layers of fixed thickness (e.g., anterior 120 μm, midcornea, and posterior 60 μm) (Spierer et al., 2016). While useful for gross comparisons, this stratification method does not align with the anatomical boundaries of the corneal natural layered structure (e.g., Bowman’s layer at ∼10% depth, Descemet’s membrane at >90% depth), potentially obscuring critical structure-function relationships at these interfaces. Therefore, a more detailed description is needed to better depict the densitometry of each layer of the cornea and reflect the microstructural information.

Our team (Li et al., 2023) had established a new layered metering method to obtained densitometry of the 50 corneal layers. Here, corneal densitometry is treated as a quantitative, *in vivo* biomarker of tissue compactness, which is influenced by the underlying microstructural organization (e.g., collagen density and packing). This novel *in vivo* approach was used to correlate structure-specific corneal integrity with macroscopic biomechanical behavior. By integrating dynamic corneal response parameters with layer-resolved densitometry measurements, this study aimed to demonstrated the first evidence of structural-functional coupling across human corneal lamellae under physiological conditions. This multimodal strategy aimed to overcome the limitations of conventional whole-cornea biomechanical assessments and provide clinically actionable insights for targeted diagnosis of layer-specific pathologies (e.g., stromal softening in keratoconus, endothelial dysfunction in glaucoma) and personalized intervention planning in refractive surgery and corneal cross-linking.

## Materials and methods

### Study design

This cross-sectional study included 221 healthy participants (221 eyes) (mean age, 27 ± 6 years, 65% female). Only the right eye was included in the analysis. The inclusion criteria were age ≥18 years, stable refraction (defined as a change in spherical equivalent [SE] ≤ 0.50 D over the past year), and a SE between −6.00 D and +3.00 D. Participants could be emmetropic or myopic. The exclusion criteria were ocular trauma and history of ophthalmic surgery, glaucoma, keratoconus, diabetes, abnormal immune function, or systemic connective tissue disease. All patients underwent complete ophthalmic examinations, including anterior segment tomography and corneal biomechanical evaluation, and provided informed consent for the use of their data. This study adhered to the tenets of the Declaration of Helsinki and was approved by the medical ethics committee of Tianjin Eye Hospital (TJYLL-2021-31).

### Corneal layered densitometry evaluation

**FIGURE 1:**
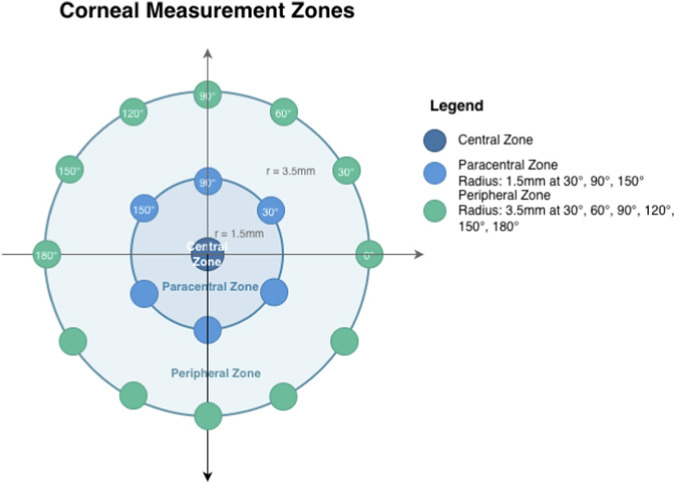
The measurement protocol. Corneal densitometry for each layer was measured from 19 predetermined points: the corneal center point (central zone), measurements at 30°, 90°, and 150° meridians at 1.5 mm radius from the corneal center (paracentral zone), and measurements at 30°, 60°, 90°, 120°, 150°, and 180° meridians at 3.5 mm radius (peripheral zone).

Corneal densitometry was obtained by Scheimpflug imaging (Pentacam-HR, Oculus GmbH, Wetzlar, Germany). In our previous study, the cornea was divided equally into 50 sublayers (the depth of each sublayer accounting for 2% of the cornea) to describe spatial patterns of corneal densitometry with depth; two densitometry peaks appeared near the Bowman membrane and Descemet membrane, while densitometry values of the stroma tends to be stable (Yang et al., 2025). In the present study, to achieve spatial correspondence between corneal densitometry and lamellar anatomical structure, an uneven layering method was adopted to reduce redundancy and improve anatomical correspondence, in which the depth near the Bowman membrane and Descemet membrane (8%–20% and 90%–100% of the corneal depth, respectively) retained a more detailed division (2% of division interval), while the depth near stroma was divided sketchy (10% of division interval). The corneal image was consequently divided into 19 layers at the following corneal depths: 0%–8%; 8%–10%; 10%–12%; 12%–14%; 14%–16%; 16%–18%; 18%–20%; 20%–30%; 30%–40%; 40%–50%; 50%–60%; 60%–70%; 70%–80%; 80%–90%; 90%–92%; 92%–94%; 94%–96%; 96%–98%; and 98%–100%. The system’s caliper function was used to manually measure corneal densitometry for each layer, with values obtained from 19 predetermined points per layer: the corneal center point (central zone), measurements at 30°, 90°, and 150° meridians at 1.5 mm radius from the corneal center (paracentral zone), and measurements at 30°, 60°, 90°, 120°, 150°, and 180° meridians at 3.5 mm radius (peripheral zone), shown as Figure 1. For each point, the densitometry measurements was recorded, and for each layer, the densitometry values from the six paracentral points and 12 peripheral points were averaged separately to represent paracentral and peripheral regional values, respectively (Li et al., 2023; Li et al., 2025); Zheng et al., 2023). Then, the curve of the densitometry of each area was plotted as a function of corneal depth. This averaging approach was used to obtain stable regional estimates, though it may not capture directional anisotropy. The sublayers with the same slope represent the same tissue density, and were reclassified as the same layer, corresponding to the specific corneal structure. All measurements were qualified; no eyelashes or eyelids were covered. The same experienced clinician (F.D.) used the same method for all measurements. Densitometry measurements were repeated by the same clinician (F.D.) on 30 randomly selected eyes after a 2-week interval. The intraclass correlation coefficient (ICC) for the average densitometry of the key anterior layer (12%–40% depth, central zone) was 0.91 (95% CI: 0.82–0.96), indicating excellent repeatability.

### Dynamic corneal response (DCR) and biomechanics evaluation

The Corvis ST visual corneal biomechanics analyzer (version 15-1902, Oculus company, Germany) was used to evaluate corneal biomechanical properties, which flattens the cornea twice by automatically ejecting pulsed airflow, and records the corneal deformation process by the Scheimpilug high-speed camera to obtain corneal dynamic response parameters, waveforms, and dynamic images, reflecting the biomechanical characteristics of the cornea calculated by internal programs. All examinations were performed with good quality scores. After instruments were calibrated, and the same experienced clinician (F.D.) performed all measurements using the same instruments.

Corneal DCR parameters included the first (A1V) and the second applanation time (A2V), the first (A1T) and the second applanation time (A2T), maximum deflection near highest concavity (DeflAmpMax), Integrated inverse concave radius (IR), stiffness parameter at the first applanation (SPA1), stiffness parameter highest concavity (SPHC), corneal biomechanical index (CBI), and maximum inverse radius (Zhang et al., 2013; Vinciguerra et al., 2016). SPA1 is the resultant pressure at the first applanation from the difference between the air-puff pressure at the corneal surface and the biomechanically corrected IOP, divided by the deflection amplitude. Higher values indicate stiffer corneas (Metzler et al., 2014; Wilson, 2020). SPHC uses the difference in DeflAmpMax minus deflection amplitude at A1 (A1DeflAmp) (Fernandez et al., 2017). A higher SPHC indicates stiffer cornea and sclera, because a stiffer sclera can limit the magnitude of maximum corneal deformation (Shen et al., 2019). IR is the integrated area under the curve of the inverse concave radius, which is the radius of curvature during the concave phase of deformation. A higher IR indicates softer tissue (Wang et al., 2023). CBI ranging from 0 (normal) to 1 (abnormal) is obtained using logistic regression with a combination of different dynamic Scheimpflug analyzer parameters. The goal is to enhance the sensitivity between keratoconic eyes and healthy eyes using a proprietary algorithm (Ni et al., 2014). Although CBI is primarily designed for keratoconus detection, it is included here as a composite biomechanical metric to explore its association with structural compactness in healthy corneas.

### Statistical analyses

Statistical analyses were performed using SPSS version 26 (IBM, Armonk, NY, USA). Descriptive statistical results included means, standard deviations, and the minimum and maximum values of the parameters. The Kolmogorov–Smirnov test was used to assess the data distribution. Correlations between DCR parameters and layered density results were analyzed using Pearson’s or Spearman’s correlation. To account for potential confounding factors, partial correlation analyses were also performed between densitometry values and DCR parameters, controlling for central corneal thickness (CCT), biomechanically corrected intraocular pressure (bIOP), age, and SE. To account for multiple comparisons across multiple corneal layers, regions, and biomechanical parameters, the significance level (α = 0.05) was adjusted using the Bonferroni correction, dividing the original α by the number of independent hypothesis tests performed. This conservative approach reduces the risk of Type I errors (false positives). The sample size was determined based on feasibility and the goal of achieving adequate power for correlation analyses. A sample size of 221 provides >80% power to detect a Pearson correlation coefficient of |r| ≥0.2 (a small-to-moderate effect) at a two-sided alpha level of 0.05, which is appropriate for this exploratory correlation study. *P* < 0.05 after Bonferroni correction was considered statistically significant.

## Results

### Basic characteristics

The mean participant age was 27 ± 6 years, and the CCT was 541.145 ± 34.373 μm. The mean IOP was 14.8 ± 2.5 mmHg (range: 11–19 mmHg). All participants were healthy with no ocular pathology or history of surgery. The average ranges and standard deviation of the biomechanical parameters are listed in Table 1.

**TABLE 1:** Basic information and corneal biomechanical parameters of participants.

Basic information	Range	Mean	Standard deviation
Age	20 to 45	27	6
CCT, μm	482 to 635	541.15	34.37
IOP, mmHg	11 to 19	14.8	2.5
A1V, m/s	0.05 to 0.23	0.12	0.07
A2V, m/s	0.33 to 0.99	0.62	0.12
A1T, ms	6.59 to 8.12	7.34	0.67
A2T, ms	18.98 to 21.10	19.28	0.95
Max inverse radius, mm^−1^	0.15 to 0.19	0.17	0.02
IR, mm^−1^	5.99 to 11.24	8.39	0.921
ARTh	387.30 to 781.93	533.81	81.21
SPA1	81.20 to 141.43	113.02	10.84
CBI	0.00 to 0.54	0.11	0.01
SPHC	9.94 to 22.94	12.37	2.24

Abbreviations: CCT, central corneal thickness; IOP, intraocular pressure; A1V, the first applanation time; A2V, the second applanation time; A1T, the first applanation time; A2T, the second applanation time; Max Inverse Radius, maximum inverse radius; IR, integrated radius; ARTh, Ambrósio’s relational thickness to the horizontal profile; SPA1, stiffness parameter in the first applanation state; CBI, corneal biomechanical index; SPHC, stiffness parameter highest concavity.

### Corneal densitometry of each corneal layer

**FIGURE 2:**
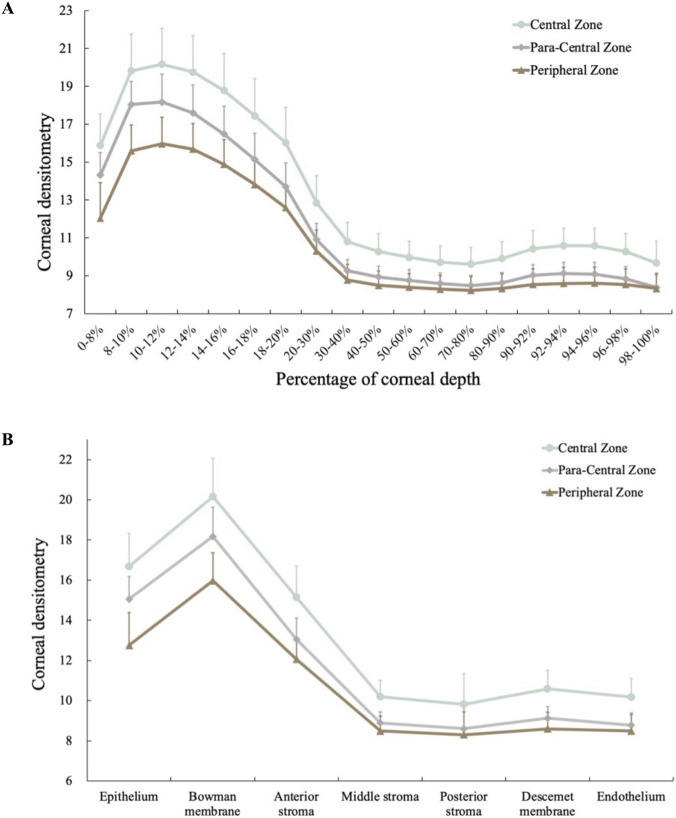
Depth-dependent densitometry curves for central, paracentral, and peripheral zones **(A)** and the layered densitometry for the seven anatomical layers **(B)**.

The depth-dependent variations in densitometry values across the 19 corneal sublayers within distinct zones are shown in Figure 2A. After merging sublayers with similar slopes, the cornea was divided into seven layers, corresponding to epithelium (0%–8% depth), Bowman membrane (8%–12%), anterior stroma (12%–40%), middle stroma (40%–80%), posterior stroma (80%–92%), Descemet membrane (92%–94%), and endothelium (94%–100%). Specifically, corneal depth densitometry from 0% to 10% showed an upward trend in the epithelium; corneal depth from 10% to 12% was regarded as the range of peak densitometry the corresponding depth was anatomically close to the Bowman membrane. Densitometry of corneal depth from 12% to 40% showed a downward trend with a similar slope, corresponding to the anterior stroma. Densitometry of 40%–80% of the corneal depth tended to be stable, corresponding to the middle stroma; densitometry values of 80%–92% of corneal depth show an upward trend with similar slopes, corresponding to the posterior stroma. Densitometry of 92%–94% of corneal depth represents the second peak, corresponding to a depth anatomically close to the Descemet membrane. Finally, 94%–100% of the corneal depth as the endothelium. Each corneal layered densitometry is shown in Figure 2B.

### Relationship between layered densitometry and corneal biomechanical parameters

Correlation analysis between layered corneal densitometry values and dynamic corneal response parameters revealed significant associations, exhibiting distinct layer-specific and regional patterns (Table 2). Overall, densitometry values in several corneal layers correlated significantly with multiple dynamic deformation metrics.

**TABLE 2:** Correlation analysis between the layered densitometry values of corneal structures and dynamic corneal response parameters.

Corneal structure	Region	Dynamic corneal response parameters									
		A1V	A2V	A1T	A2T	DeflAmpMax	IR	SPA1	SPHC	Max inverse radius	CBI
Epithelium	Central	−0.019	−0.049	−0.039	−0.065	0.021	0.014	−0.137*	−0.095	0.014	0.126
	Paracentral	0.007	−0.087	−0.075	−0.101	0.049	0.049	−0.149**	−0.121*	0.019	0.091
	Peripheral	0.002	−0.153*	0.023	−0.084	0.057	0.018	−0.036	−0.052	0.015	−0.005
Bowman’s membrane	Central	−0.210**	0.149**	0.158**	−0.191**	−0.138*	−0.170**	0.196**	0.193**	−0.093	−0.150**
	Paracentral	−0.178**	0.050	0.139**	−0.201**	−0.103	−0.141*	0.184**	0.172*	−0.085	−0.167**
	Peripheral	−0.141**	−0.005	0.176**	−0.147*	−0.080	−0.153**	0.240***	0.199***	−0.099	−0.219***
Anterior stroma	Central	−0.251***	0.239***	0.235***	−0.159**	−0.215***	−0.229***	0.296***	0.300***	−0.143**	−0.142**
	Paracentral	−0.256***	0.182**	0.295***	−0.169**	−0.235**	−0.248***	0.325***	0.329***	−0.138**	−0.096
	Peripheral	−0.100	0.012	0.122	−0.045	−0.037	−0.134*	0.193***	0.142**	−0.133**	−0.071
Middle stroma	Central	−0.020	−0.007	−0.045	−0.012	0.026	−0.049	−0.026	−0.034	−0.107	−0.04
	Paracentral	−0.100*	0.023	0.052	−0.120	−0.068	−0.082	0.078	0.067	−0.072	−0.103
	Peripheral	−0.070	−0.045	0.135**	−0.112	−0.062	−0.089	0.152**	0.123*	−0.021	−0.167**
Posterior stroma	Central	−0.130	0.062	0.069	−0.139*	−0.067	−0.093	0.102	0.064	−0.051	−0.028
	Paracentral	−0.105	−0.007	0.051	−0.165**	−0.042	−0.065	0.143*	0.052	−0.044	−0.063
	Peripheral	−0.084	−0.071	0.119	−0.127	−0.025	−0.085	0.074	0.099	−0.052	−0.138*
Descemet’s membrane	Central	−0.114	0.075	0.066	−0.061	−0.052	−0.118	0.163**	0.085	−0.140**	−0.114
	Paracentral	−0.108	−0.005	0.061	−0.132*	−0.065	−0.094	0.143*	0.082	−0.061	−0.159**
	Peripheral	−0.038	−0.091	0.094	−0.070	−0.019	−0.025	0.074	0.069	0.039	−0.097
Endothelium	Central	−0.089	0.053	0.032	−0.081	−0.06	−0.055	0.149*	0.084	−0.052	−0.12
	Paracentral	−0.122	−0.038	0.034	−0.137*	−0.043	−0.101	0.221***	0.092	−0.09	−0.184**
	Peripheral	−0.031	−0.117	0.065	−0.065	−0.003	−0.016	0.007	0.057	0.037	−0.086

^*^ *P* < 0.05 after Bonferroni correction;

^**^ *P* < 0.01 after Bonferroni correction, and

^***^ *P* < 0.001 after Bonferroni correction.

Abbreviations: A1V, the first applanation time; A2V, the second applanation time; A1T, the first applanation time; A2T, the second applanation time; DeflAmpMax, maximum corneal deviation amplitude; IR, inverse radius; SPHC, stiffness parameter highest concavity; Max Inverse Radius, maximum inverse radius; SPA1, stiffness parameter in the first applanation state; CBI, corneal biomechanical index.

The most significant and consistent correlations were observed in the anterior corneal structures, particularly Bowman’s membrane and the anterior stroma. In the central region, increased densitometry in both Bowman’s membrane and the anterior stroma demonstrated significant positive correlations with parameters indicative of corneal stiffness (SPA1 and SPHC: Bowman’s central SPA1 r = 0.196, SPHC r = 0.193, both *P* < 0.01; Anterior stroma central SPA1 r = 0.296, *P* < 0.05, SPHC r = 0.300, *P* < 0.05). Conversely, densitometry in these layers showed significant negative correlations with parameters related to deformation susceptibility or timing in the central region (e.g., Anterior stroma central: A1V r = −0.251, *P* < 0.05, DeflAmpMax r = −0.215, *P* < 0.05, IR r = −0.229, *P* < 0.001). This pattern of positive correlations with stiffness parameters and negative correlations with deformation/time parameters was largely maintained in the paracentral region for these anterior layers but diminished in the periphery. Notably, the correlations between anterior stromal densitometry and stiffness parameters (SPA1, SPHC) in the central and paracentral regions were the most significant observed across all layers and regions.

Correlations involving other corneal layers (epithelium, mid-stroma, posterior stroma, Descemet’s membrane, endothelium) were generally weaker and more spatially restricted or parameter-specific. Epithelial densitometry showed a weak negative correlation with SPA1 in the paracentral region (r = −0.149, *P* < 0.01) and with A2V in the periphery (r = −0.153, *P* < 0.001). Mid-stromal densitometry correlated negatively with A1V in the paracentral region (r = −0.1, *P* < 0.05) and positively with stiffness parameters SPA1/SPHC in the periphery (SPA1 r = 0.152, *P* < 0.01, SPHC r = 0.123, *P* < 0.05), while also correlating negatively with CBI peripherally (r = −0.167, *P* < 0.01). Significant correlations for the posterior stroma, Descemet’s membrane, and endothelium were sparse and isolated to specific regions and parameters (e.g., Posterior stroma paracentral A2T: r = −0.165, *P* < 0.01; Descemet’s membrane central SPA1: r = 0.163, *P* < 0.01; Endothelium paracentral SPA1: r = 0.221, *P* < 0.001, and CBI: r = −0.184, *P* < 0.01). In contrast, central mid-stromal and central posterior stromal densitometry showed no statistically significant correlations with the majority of biomechanical parameters examined.

After adjusting for CCT, bIOP, age, and SE in supplementary partial correlation analyses, the primary layer-specific associations reported above remained consistent. For instance, the correlation between anterior stromal densitometry (central zone) and SPA1 remained positive and significant (partial r = 0.268, *P* < 0.001), only slightly attenuated from the unadjusted correlation (r = 0.300). Similarly, the negative association between anterior stromal densitometry and IR persisted after adjustment (partial r = −0.207, *P* = 0.001; unadjusted r = −0.229). The correlations involving Bowman’s membrane densitometry and stiffness parameters also retained significance in the adjusted model (e.g., central Bowman’s densitometry vs. SPHC: partial r = 0.18, *P* = 0.004).

## Discussion

This study established links between depth-resolved corneal structural compactness and macroscopic biomechanical behaviors in the healthy human. By integrating a novel, anatomically-informed layered densitometry with dynamic deformation analysis, which reflects the biomechanical properties of the cornea, we demonstrated that regional light backscatter variations are significantly associated with stress-response behavior. This work provided *in vivo* evidence supporting long-held *ex vivo* concepts of corneal structure-function relationships and offers a new paradigm for clinical assessment.

A key finding is the significant positive correlation between anterior-most sublayer densitometry (10%–40% depth) and overall corneal stiffness measured by dynamic deformation analysis. This correlation are not merely attributable to variations in CCT, IOP, age, or refractive error within our cohort. The densitometry peak in this anterior-most conea represented two intimately linked structures: Bowman’s layer and the anterior stroma. Bowman’s layer is an 8–12 µm thick, acellular lamina composed of fine collagen fibrils (Types I, III, V) with diameters in the range of 20–30 nm, significantly smaller than those in the deeper stroma (Wilson, 2020). These fibrils form a densely packed, randomly interwoven felt-like sheet. This high-density randomness at light-comparable scales increases light scattering, manifesting as high Scheimpflug densitometry (Komai and Ushiki, 1991). Immediately posterior to Bowman’s layer, the anterior stroma lamellae demonstrate extensive branching and interweaving. It is this anterior third of the stroma that is widely recognized as the corneal primary structural element (Scarcelli et al., 2012). Previous study using X-ray scattering and electron microscopy has revealed that many of anterior lamellae insert obliquely into the posterior aspect of Bowman’s layer, creating a highly integrated, mechanically robust composite structure (Winkler et al., 2011). This complex isotropic arrangement provides exceptional shear resistance, explaining the anterior stroma’s greater tensile strength and higher elastic modulus versus posterior stroma (Winkler et al., 2011; Esporcatte et al., 2020; Yang et al., 2022).

The observed microstructural pattern may help interpret our biomechanical associations: higher densitometry in anterior layers was associated with a stiffer corneal response, such as a higher SP-A1 and a lower overall deformation amplitude. Mechanistically, the interwoven anterior complex serves as the cornea’s primary structural backbone, resisting biomechanical analyzer air-puff forces from the biomechanical analyzer (Salouti et al., 2020). Stromal lamellae anchoring into Bowman’s rigid layer creates a shear/tension-resistant composite material. Thus, the anterior densitometry peak is not merely an optical reading but an *in vivo* biomarker of the cornea’s principal load-bearing integrity.

A distinct posterior densitometry peak consistently occurred in the corneal deepest 8%, aligning with Descemet’s membrane. This finding reflects fundamental microstructural differences: Descemet’s membrane is an endothelial-secreted basement membrane composed of non-lamellar collagen IV/VIII meshwork, creating a sharp refractive interface with posterior stroma (de Oliveira and Wilson, 2020). Biomechanically, while bulk models historically dismissed Descemet’s membrane’s contribution (Petsche et al., 2012), atomic force microscopy reveals its exceptionally high Young’s modulus [potentially exceeding Bowman’s layer (Zhang et al., 2013)]. Our data indicate Descemet’s membrane’s densitometry-reflected structural state minimally modulates whole-corneal deformation in health. Pathologically, however, increased posterior densitometry may signal structural degradation and weaker mechanics (Zheng et al., 2023).

Between the boundary peaks, most stromal densitometry (10%–90% depth) remained relatively stable and low—corresponding to the corneal transparency-optimized region. From a biomechanical standpoint, while the anterior cornea governs initial deformation resistance, the posterior structures may dominate viscoelastic recovery (Almubrad and Akhtar, 2011; Madl and Myung, 2021). The posterior stroma itself is characterized by thicker, wider, and more parallel-oriented lamellae with less interweaving (Almubrad and Akhtar, 2011; Madl and Myung, 2021), which is thought to facilitate greater inter-lamellar slippage, whereby friction generated by parallel lamellae sliding through the proteoglycan-rich hydrated matrix constitutes the key mechanism for viscoelastic energy dissipation in the cornea. Subtle increases in stromal densitometry could signify stromal disruptions, such as stromal edema or changes in proteoglycan composition, which would in turn be expected to alter viscoelastic properties. Future analyses should correlate posterior densitometry with recovery-phase parameters like corneal hysteresis.

A principal methodological contribution of this study is the implementation of an anatomically-informed, high-resolution layered densitometry model. Standard clinical densitometry typically provides values for the entire corneal thickness or for broad anterior, central, and posterior segments. This approach averages out depth-dependent variations in corneal structure. By designing an uneven 19-layer model with high resolution near the anterior and posterior boundaries, we spatially aligned optical analysis with corneal anatomical layers, unmasking critical boundary-layer contributions otherwise obscured.

Built upon previous efforts (Yang et al., 2025), we provided a method to “see” the biomechanical implications of anatomical layers without the need for invasive sampling, bridging the gap between the microscopic world of tissue compactness and clinical practice. The layer-specific associations identified here suggested a potential clinical utility. For example, in keratoconus, known to involve disruption of Bowman’s layer and anterior stromal weakening (Ma et al., 2018), a combined assessment of anterior densitometry patterns and biomechanical indices [like lowered SPA1 and elevated CBI (Huo et al., 2024)]. might enhance early detection sensitivity, a hypothesis requiring prospective validation. Similarly, postoperative monitoring of layered densitometry (e.g., tracking haze-related spikes) alongside biomechanical parameters could provide a more integrated view of healing after refractive surgery (He et al., 2023; Cao et al., 2023; Xia et al., 2016).

This study is not without limitations. Its cross-sectional nature precludes causal inference, requiring longitudinal validation. Densitometry remains an indirect microstructure proxy measuring light backscatter, not direct collagen orientation or nano-mechanical properties. Furthermore, corneal hydration alters densitometry by changing the refractive index mismatch between collagen fibrils and the matrix. Although our cohort included only healthy individuals without signs of corneal edema, hydration was not controlled, introducing variability. Future studies using hydration-sensitive metrics like confocal microscopy could clarify this effect. Finally, while establishing young healthy norms, future applications must extend to older populations.

In summary, this study provides compelling evidence that depth-resolved corneal densitometry is a valuable *in-vivo* biomarker for the underlying anatomical layers that dictates corneal biomechanics. It revealed that anterior stromal compactness was the primary layer-associated factor of corneal stiffness. Higher densitometry in anterior layers was associated with a stiffer corneal response, such as a higher SP-A1 and a lower overall deformation amplitude. In contrast, Desceme’s membrane’s densitometry-reflected structural state showed minimal association with whole-corneal deformation. Establishing the links between optical signature and dynamic deformation parameters helps pave the way for a more holistic and powerful clinical assessment of corneal health, holding significant promise for improving the diagnosis and management of a wide spectrum of corneal disorders.

## Data Availability

The datasets presented in this study can be found in online repositories. The names of the repository/repositories and accession number(s) can be found in the article/supplementary material.
